# Supplementary material for: Pan-Cancer Analysis Identifies Tumor Cell Surface Targets for CAR-T Cell Therapies and Antibody Drug Conjugates
Source: Cancers (Basel). 2022 Nov 18;14(22):5674. doi: 10.3390/cancers14225674 (PMC9688665; doi:10.3390/cancers14225674)
Supplement: Supplementary file 1 [file cancers-14-05674-s001.zip › Supplementary Information.doc.pdf]

Supplementary Figure/Table

Figure S1

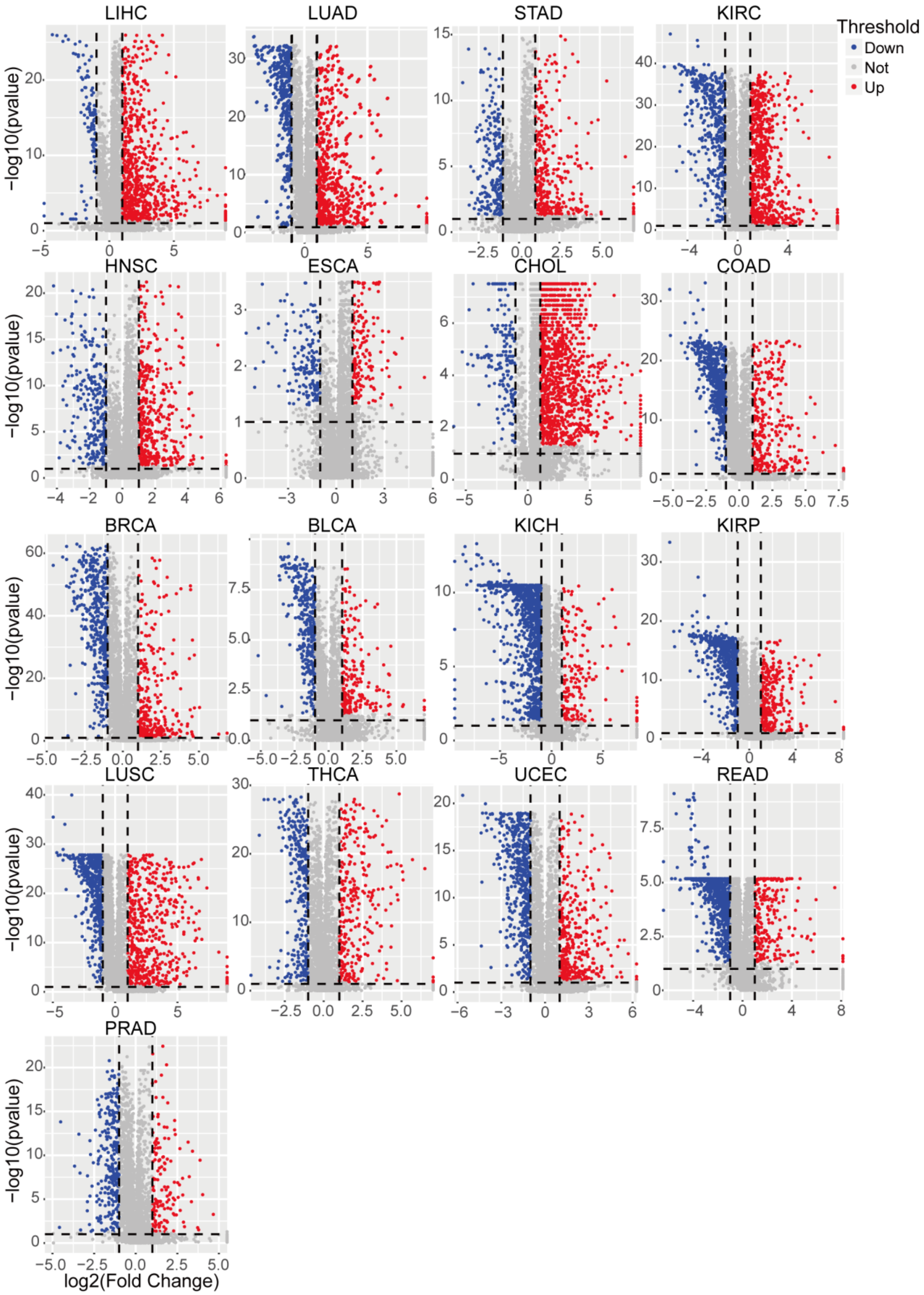

Figure S1. Analysis of the differential expression of membrane

protein-coding genes.

The volcano plot showed the differential expression analysis of 3919 membrane protein-coding genes in 17 cancers. The red dots indicated the genes up-regulated in tumor tissues. The blue dots indicated the genes down-regulated in tumor tissues. The gray dots indicated the genes not significantly changed. The FoldChange of Tumor/Normal>2, and adjusted p value<0.01 was considered statistically significant.

Figure S2

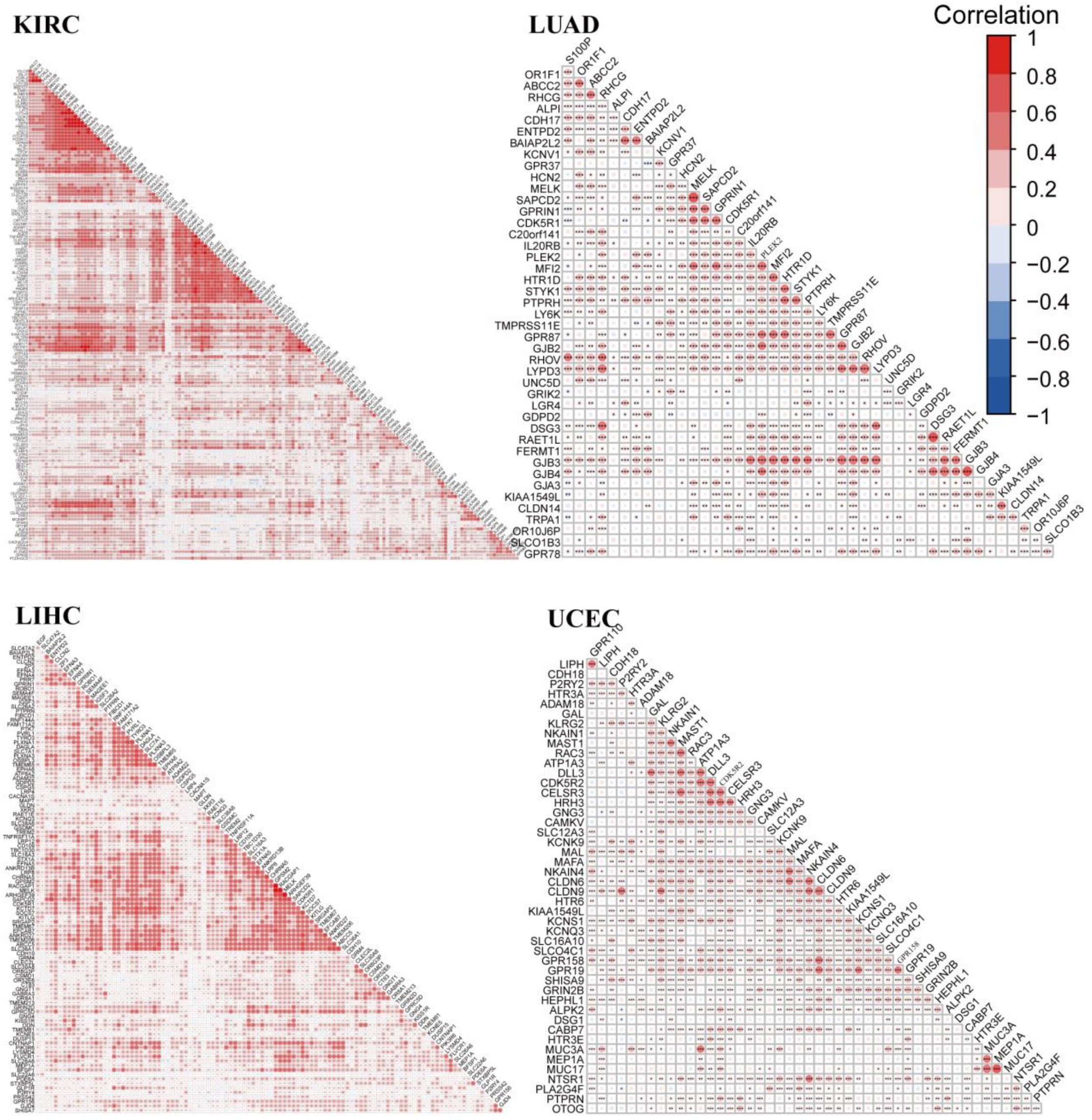

Figure S2. *Pearson* correlation analysis of the significantly high-expression and high-risk membrane protein-coding genes in some tumor tissues in TCGA.

Figure S3

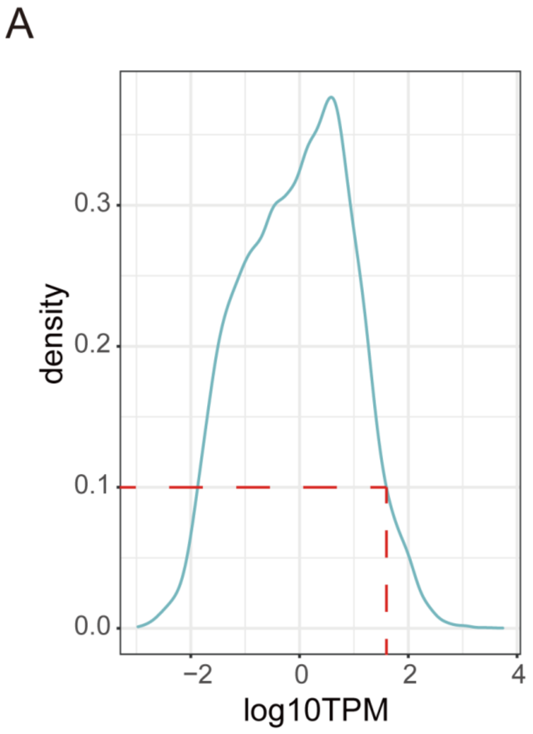

Figure S3. Cumulative distribution of the expression of 371 candidate membrane proteins. The red dashed line was determined to separate the differential expression patterns of these genes.

Table S1. The information on the results of HR, FC, Correlation and Prognosis of individual or paired genes.

Table S2. Patients' risk scores calculated for gene pairs based on the risk score system.
