# Supplementary figures and images for: Pan-Cancer Analysis Identifies Tumor Cell Surface Targets for CAR-T Cell Therapies and Antibody Drug Conjugates
Source: Cancers (Basel). 2022 Nov 18;14(22):5674. doi: 10.3390/cancers14225674 (PMC9688665; doi:10.3390/cancers14225674)

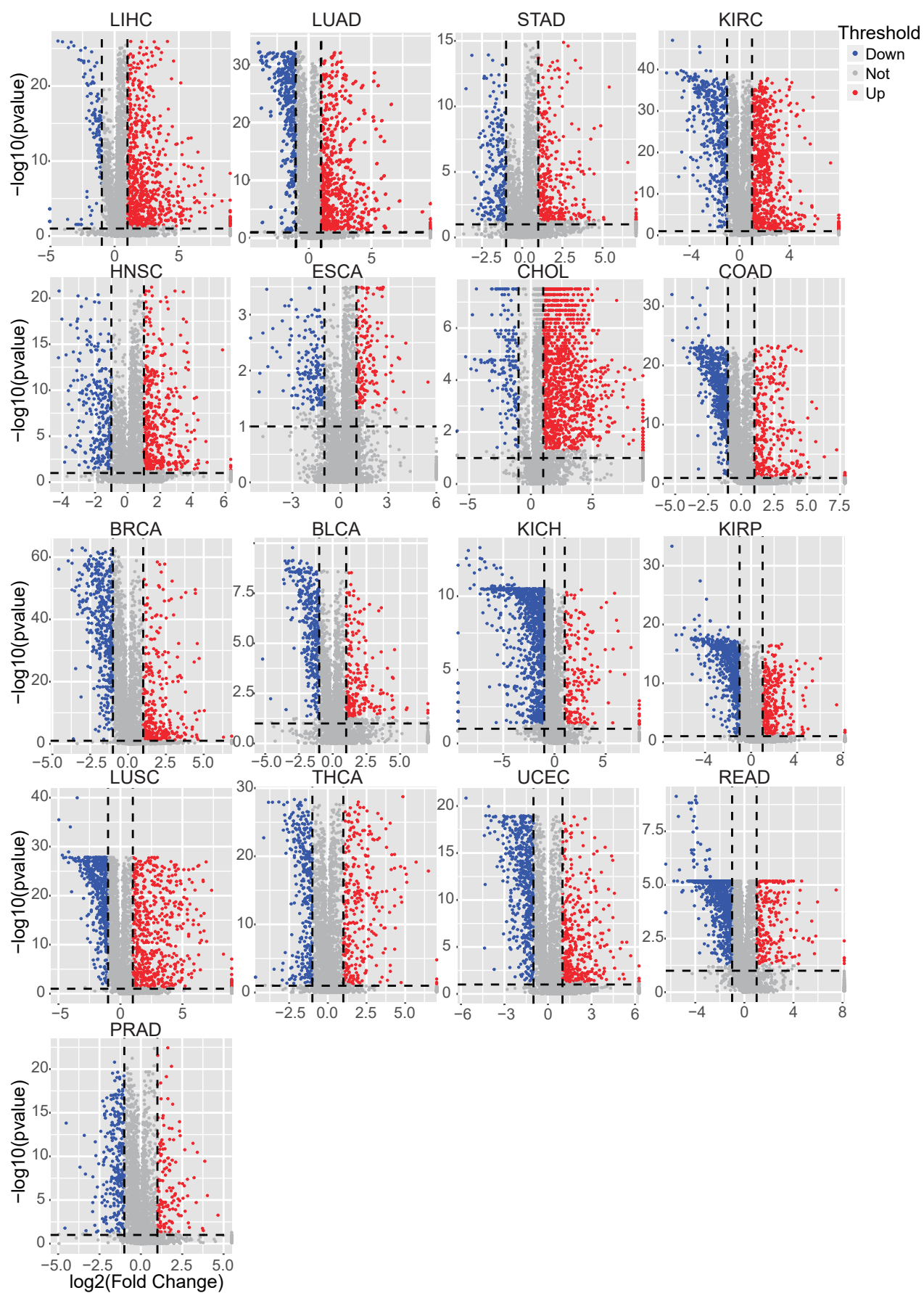

Supplement: Supplementary file 1 [file cancers-14-05674-s001.zip › Figure S1.pdf]

# KIRC

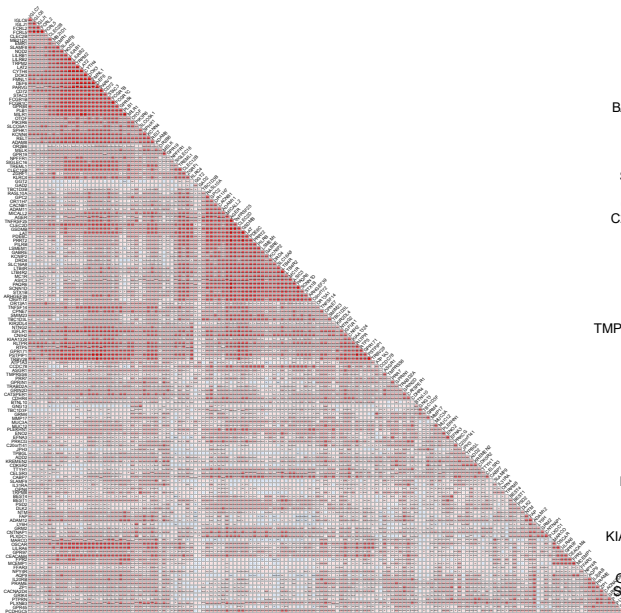

# LUAD

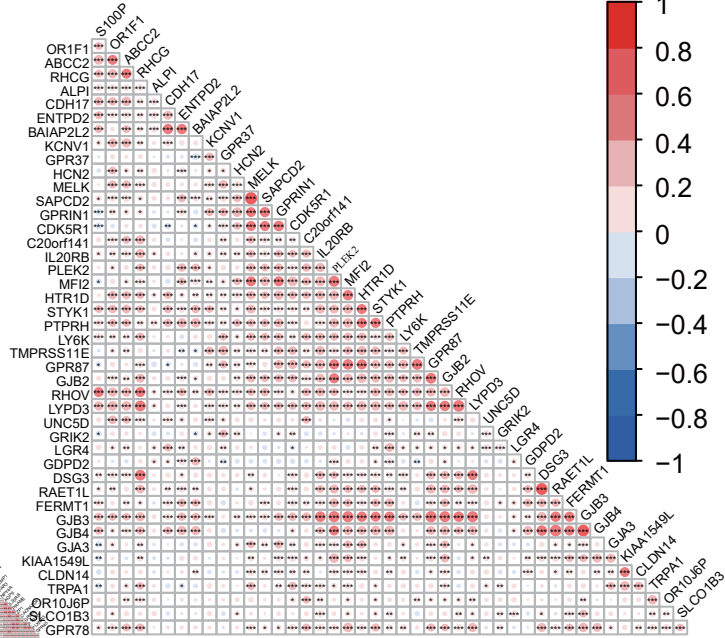

Correlation

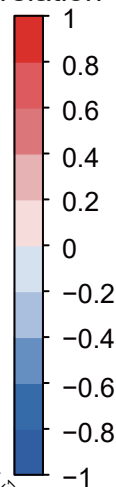

# LIHC

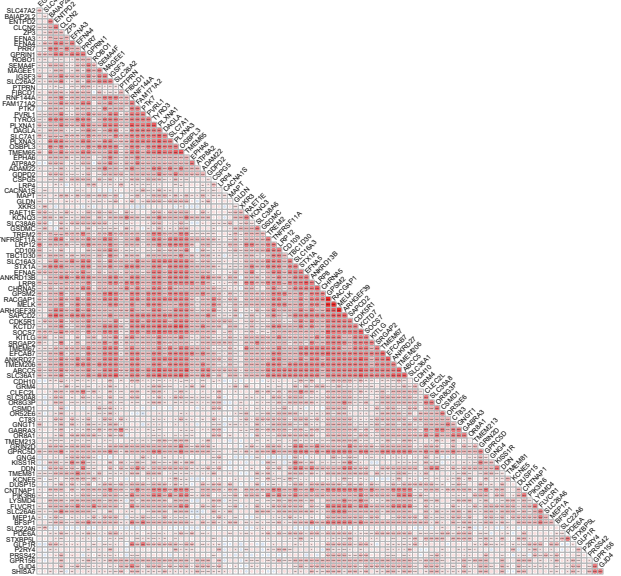

# UCEC

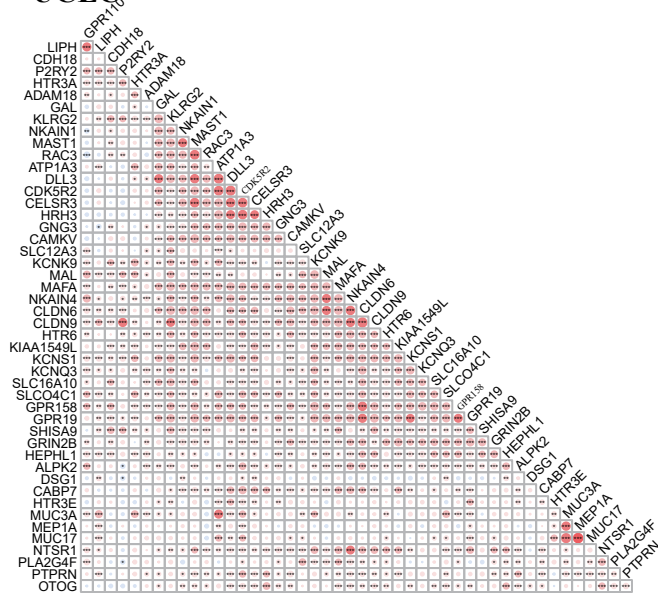

Supplement: Supplementary file 1 [file cancers-14-05674-s001.zip › Figure S2.pdf]

A

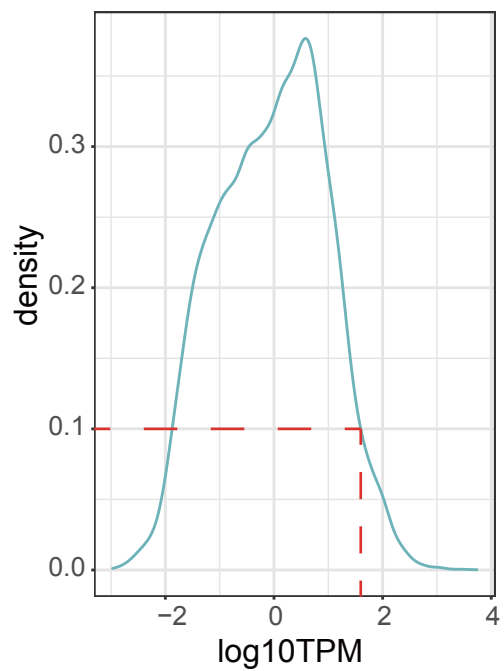

Supplement: Supplementary file 1 [file cancers-14-05674-s001.zip › Figure S3.pdf]
